# A Generalized Hamilton Robust Control Scheme of Trajectory Tracking for Intelligent Vehicles

**DOI:** 10.3390/s23156975

**Published:** 2023-08-05

**Authors:** Yu Zhang, Wenhui Pei, Qi Zhang, Baosen Ma

**Affiliations:** 1 School of Information Science and Electrical Engineering, Shandong Jiaotong University, Jinan 250357, China; zhangyu8518@163.com (Y.Z.);; 2 School of Control Science and Engineering, Shandong University, Jinan 250061, China

**Keywords:** intelligent vehicles, generalized Hamilton theory, trajectory tracking, robust control

## Abstract

To ensure the accuracy and stability of intelligent-vehicle-trajectory tracking, a robust trajectory-tracking control strategy based on generalized Hamilton theory is proposed. Firstly, a dynamic Hamilton dissipative controller (DHDC) and trajectory-tracking Hamilton dissipative controller (TTHDC) were designed based on the established vehicle-dynamics control system and trajectory-tracking control system using the orthogonal decomposition method and control-switching method. Next, the feedback-dissipative Hamilton realizations of the two systems were obtained separately to ensure the convergence of the system. Secondly, based on the dissipative Hamilton system designed by TTHDC, a generalized Hamilton robust controller (GHRC) was designed. Finally, the co-simulation of Carsim and MATLAB/Simulink was used to verify the effectiveness of the three control algorithms. The simulation results show that DHDC and TTHDC can achieve self-stabilizing control of vehicles and enable certain control effects for the trajectory tracking of vehicles. The GHRC solves the problems of low tracking accuracy and poor stability of DHDC and TTHDC. Compared with the sliding mode controller (SMC) and linear quadratic regulator (LQR) controller, the GHRC can reduce the lateral error by 84.44% and the root mean square error (RMSE) by 83.92%, which effectively improves the accuracy and robustness of vehicle-trajectory tracking.

## 1. Introduction

As a result of research into and application of a series of scientific technologies, such as autonomous driving, 5G networks, and vehicle-to-everything communication, cars are gradually evolving from a single mode of transportation and are being incorporated into an increasing number of life scenarios, moving towards intelligentization [1]. Intelligent vehicles have significant advantages in improving driving safety and reducing traffic accidents, making their development a major research topic worldwide [2]. As one of the core issues in intelligent vehicles, trajectory-tracking control is divided into lateral and longitudinal control [3]. Precise control over the lateral motion of a vehicle can affect the safety, comfort, and economy of its driving process [4]. However, the highly nonlinear dynamics characteristics and parameter uncertainties of vehicle systems increase the complexity of dynamic control. Therefore, the design and verification of reasonable trajectory-tracking-control strategies have significant research significance.

From the perspective of control algorithms, trajectory-tracking control technology needs to balance the accuracy and stability of tracking. Currently, common control methods include PID control, sliding mode controllers (SMC), model predictive control (MPC), linear quadratic regulator (LQR) control, and fuzzy control [5,6]. Specifically, the PID control method has a wide range of applications and has the advantages of a simple structure and a fast response speed. The traditional PID methods may require different PID parameters for different models or path conditions to achieve stable control. In comparative terms, some improved PID methods may achieve better control results, such as: fuzzy-PID, neural network adaptive PID, Q-learning adaptive PID, etc. The SMC has strong robustness and can respond quickly to changes in the system, but the discontinuity of the control may cause system oscillation, and it requires the full state information of the system, which may increase the complexity of the system. The MPC can handle multiple-input–multiple-output systems and has the ability to predict the future states of the system, but it requires the online solution of optimization problems, which may increase computational complexity and make parameter tuning difficult. Using LQR control, one can directly obtain the controller by solving the Riccati equation, which is computationally simple and fast, but the design of the LQR controller depends on the model equation of the controlled object. Therefore, it may not work well when the system’s model error is large. Fuzzy control does not require accurate system models and has strong fault tolerance. It can effectively control complex systems, but it requires the manual design of fuzzy rules and fuzzy sets, which may be difficult. Therefore, when choosing a control strategy, a trade-off should be made based on the specific situation [7].

In recent years, improvements to traditional control algorithms in the field of autonomous driving have been studied. Bin Zhao et al. [8] applied a genetic algorithm to design a trajectory-tracking PID controller for autonomous ground vehicles. This method may have slightly lower control accuracy and poorer robustness than some advanced controllers. Yanxin Nie et al. [9] proposed an adaptive spiral sliding mode controller to address the chattering problem in traditional sliding mode controllers. Although this controller demonstrated superior tracking performance, it may require a significant amount of computational resources and time for parameter optimization and controller design. Haidong Wu et al. [10] designed a linear time-varying MPC to reduce computational complexity, which locally linearizes the nonlinear vehicle model at each sampling point. However, this method may have limitations in tracking accuracy for complex road conditions or multi-vehicle cooperative driving, as it requires a high level of understanding and mastery of the algorithm. Zhejun Huang et al. [11] established a prediction model using backward Euler integration. This paper mainly studies the calculation error generated in the discretization processes of nonlinear systems, which has certain guiding significance for the design of MPC controllers.

With the development and improvement of intelligent driving technology, the trajectory-tracking-performance requirements of intelligent vehicles are constantly increasing, and traditional control methods can no longer meet these demands. The emergence of new control algorithms provides various solutions to the trajectory-tracking problem of intelligent vehicles. For instance, Kaleb Ben Naveed et al. [12] proposed a robust hierarchical reinforcement learning (HRL) framework for learning autonomous driving policies. Yassine Kebbati et al. [13] presented an adaptive-parameter PID trajectory-tracking algorithm based on reinforcement learning and curvature feedforward control. Yassine Kebbati et al. [14] also proposed an adaptive LPVMPC autonomous driving controller optimized by the genetic algorithm and a neural network. Dongdong Yuan et al. [15] combined the dynamic characteristics of autonomous vehicles and proposed an improved model-free adaptive control algorithm. However, adaptive control algorithms are sensitive to initial conditions, have high computational complexity, and may lead to poor real-time performance. In addition, the slow convergence of parameters may result in performance degradation in the control system if the parameter estimation is inaccurate. The use of reinforcement learning, neural networks, and other methods may also lead to problems such as long training times, unstable training processes, high model complexity, and poor interpretability. It is worth noting that vehicle-planning control strategies that combine a path-planning algorithm that generates trajectories through real-time sensing with trajectory-tracking control also achieve great results. Runqi Chai et al. [16] proposed a control framework that integrates real-time trajectory planning and tracking. A motion planner was designed based on a recurrent DNN-based (RDNN) method and then combined with two migration learning strategies to achieve real-time generation of parking trajectories for different AGVs. Next, the trajectory-tracking control of the vehicle was accomplished based on the designed adaptive learning NN-based (ALNN) control algorithm. Hye Young An et al. [17] proposed a real-time path planning and trajectory-tracking control algorithm to generate the next position in real time using various sensors to obtain information about the detected lanes, and then the steering angle was calculated using a pure tracking algorithm based on the generated position, so that the algorithm can effectively adapt to real-time driving at different speeds.

Generalized Hamilton control systems are extensions of traditional Hamilton systems, which are dynamic systems. This type of system possesses clear structure and well-defined physical meanings, and exhibits structural integrity [18]. The Hamilton function (total energy of the system) is its quasi-Lyapunov function, which shows significant advantages in stability analysis, stabilization control, and other issues [19]. In addition, the Hamilton control algorithm is easy to apply and requires relatively few parameter adjustments. Currently, Hamilton system-based methods have been widely used in the control of power systems and mechanical systems, but have rarely been applied in the field of autonomous driving. Li et al. [20] proposed a Hamilton control algorithm to address the control problem in the integrated chassis system of electric vehicles steering and suspension. They designed a Hamilton controller for the nonlinear integrated model of the vehicle’s steering and suspension, but it was not applied to trajectory-tracking control. In a study by Chen et al. [21], based on Hamilton theory and combined with tire force optimization, the layered control of path tracking for unmanned four-wheel steering vehicles was realized, which has a certain tracking effect. However, the article did not consider problems such as stability control and model uncertainty during the vehicle driving processes, did not perform a rigorous derivation of the generalized Hamilton realization of the trajectory-tracking-control system for intelligent vehicles, and did not perform a comparative analysis with different control methods.

In this paper, a new intelligent vehicle trajectory-tracking control method based on the generalized Hamilton theory is proposed. The trajectory-tracking control of intelligent vehicles is realized by using the advantages of the complete and clear structure of the generalized Hamilton control system; fewer parameters need to be adjusted, and it has better stability.

The contributions of this work can be summarized as follows:

- A trajectory-tracking control method based on a generalized Hamilton theory is proposed. According to the two system models commonly used in trajectory-tracking control of intelligent vehicles, a dynamic Hamilton dissipative controller (DHDC) and a trajectory-tracking Hamilton dissipative controller (TTHDC) are designed to obtain the feedback-dissipative Hamilton realizations of intelligent vehicle systems;
- Based on the feedback-dissipative Hamilton realization of vehicle systems obtained via TTHDC, a generalized Hamilton robust controller (GHRC) is designed for the trajectory-tracking control of intelligent vehicles in conjunction with a Hamilton robust control principle, which in turn solves some problems of DHDC and TTHDC;
- According to the simulation results, under the same simulation environment, compared with the traditional sliding mode controller and LQR controller, the designed GHRC can reduce the lateral error by a maximum of 84.44% and the root mean square error (RMSE) by 83.92%, which effectively improves the accuracy and robustness of vehicle-trajectory tracking and lays the foundation for subsequent further research.

## 2. Modeling of Intelligent Vehicle Systems

### 2.1. The Vehicle Dynamics Model

**Figure 1:**
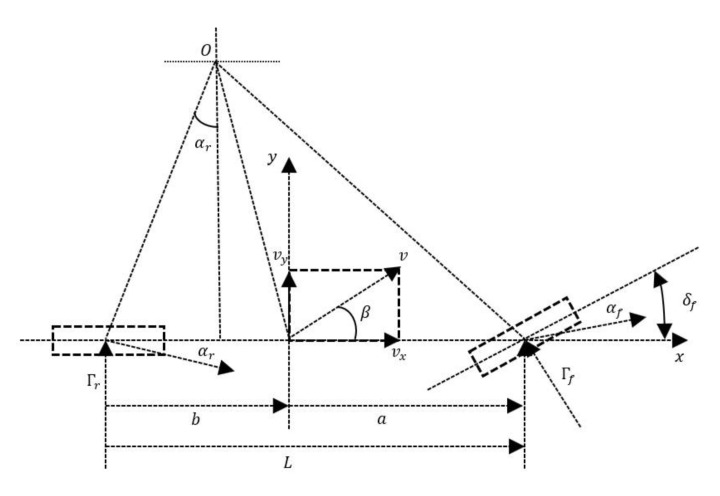
Two degree of freedom vehicle dynamics model.

The vehicle dynamics model is shown in Figure 1. It is based on reasonable assumptions and simplifications that neglect the effects of the vehicle’s steering system, suspension system and aerodynamics.

The integration of the system model shown in Figure 1 and mechanical equations lead to a major result—the derivation of the dynamic equations of intelligent vehicles [22]. An essential assumption in this derivation is the constant longitudinal velocity $v_{x}$.

(1) $$ma_{y}=\Gamma_{f}cos\delta_{f}+\Gamma_{r}$$

(2) $$I_{z}\dot{\gamma }=a\Gamma_{f}cos\delta_{f}-b\Gamma_{r}$$

where m, $a_{y}$, $\delta_{f}$, γ, and $I_{z}$, respectively represent the mass, lateral acceleration, FWSA, yaw rate and moment of inertia around z-axis of vehicles. $\Gamma_{f}$, $\Gamma_{r}$, a and b represent the lateral forces on the front and rear wheels and the distance to the center of mass of vehicles, respectively.

When $\delta_{f}$ is tiny ($cos\delta_{f}\approx 1$), the lateral forces $\Gamma_{f}$ and $\Gamma_{r}$ can be approximately expressed as the product of the side deflection stiffness ($K_{f}$, $K_{r}$) and the side deflection angle ($\alpha_{f}$, $\alpha_{r}$) of the front and rear wheels, respectively. Namely, the lateral tire force can be expressed as follows:

(3) $$\Gamma_{f}=K_{f}\alpha_{f}$$

(4) $$\Gamma_{r}=K_{r}\alpha_{r}$$

Based on the above vehicle model and combined with the rigid body kinematics of velocity synthesis and decomposition, the relationship between the $a_{y}$ and lateral displacement y of vehicles, as well as the specific expressions of $\alpha_{f}$ and $\alpha_{r}$ can be obtained.

(5) $$\left\{\begin{array}{l} v_{y}=\dot{y}\\ a_{y}=\ddot{y}+v_{x}\dot{\psi }\\ a_{r}=\frac{v_{y}-b\dot{\psi }}{v_{x}}\\ a_{f}=\frac{a\dot{\psi }+v_{y}}{v_{x}}-\delta_{f} \end{array}\right.$$

In the equation, ψ and $v_{y}$ are the heading angle and lateral speed of vehicles, respectively.

According to the above formula, these can be rearranged to obtain [23]:

(6) $$\left[\begin{array}{c} \ddot{y}\\ \ddot{\psi } \end{array}\right]=\left[\begin{array}{cc} \frac{K_{f}+K_{r}}{mv_{x}} & \frac{{aK}_{f}-bK_{r}}{mv_{x}}-v_{x}\\ \frac{{aK}_{f}-bK_{r}}{I_{z}v_{x}} & \frac{{a^{2}K}_{f}+b^{2}K_{r}}{I_{z}v_{x}} \end{array}\right]\left[\begin{array}{c} \dot{y}\\ \dot{\psi } \end{array}\right]+\left[\begin{array}{c} -\frac{K_{f}}{m}\\ -\frac{aK_{r}}{I_{z}} \end{array}\right]\delta_{f}$$

### 2.2. The Trajectory Tracking Model

**Figure 2:**
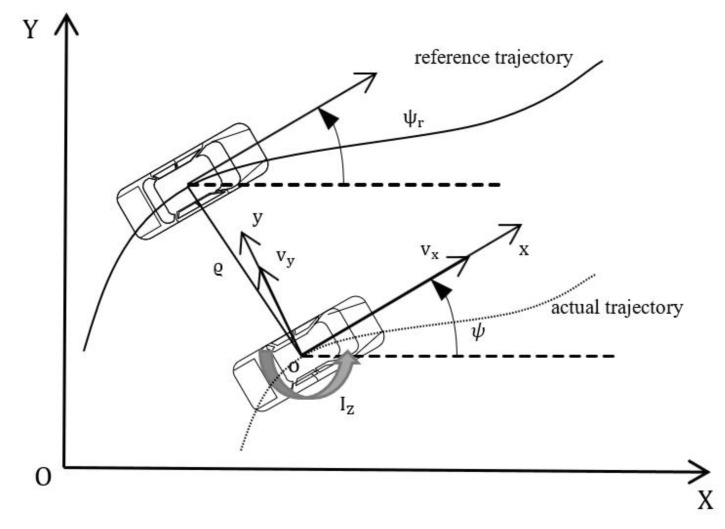
Vehicle trajectory tracking model.

To better meet the requirements of accuracy, stability and adaptability in lateral control of intelligent vehicles and to characterize the vehicle’s motion characteristics during actual driving, we have established a trajectory tracking model as shown in Figure 2.

To achieve the goal of trajectory-tracking control, we performed model analysis and formula derivation on Figure 2 to obtain the following equations:

(7) $$\left\{\begin{array}{l} \varrho_{\psi }=\psi -\psi_{r}\\ {\dot{\varrho }}_{\psi }=\dot{\psi }-{\dot{\psi }}_{r}\\ \dot{\psi }=\gamma \\ {\dot{\psi }}_{r}=v_{x}\rho \\ \dot{\varrho }=v_{x}\sin\psi +v_{y}\cos\psi \end{array}\right.$$

In the equation, $\varrho_{\psi }$ represents the heading error of vehicles and $\psi_{r}$ represents the desired heading angle. ρ represents the curvature of the desired trajectory; ϱ represents the lateral error.

Let $\xi ={\left[\begin{array}{cccc} \xi_{1} & \xi_{2} & \xi_{3} & \xi_{4} \end{array}\right]}^{T}={\left[\begin{array}{cccc} \varrho & \dot{\varrho } & \varrho_{\psi } & {\dot{\varrho }}_{\psi } \end{array}\right]}^{T}$, ${\mu =\delta }_{f}$, substituting Equation (7) into Equation (6) and simplifying yield the control system equation for vehicle-trajectory tracking:

(8) $$\dot{\xi }=A\xi +B\mu +C\omega$$

In the equation, A, B, and C are coefficient matrices; ξ, μ represents the state variable and control variable, respectively; ω denotes the interference of the desired heading angle on the system.

$$A=\left[\begin{array}{cccc} 0 & 1 & 0 & 0\\ 0 & a_{1} & a_{2} & a_{3}\\ 0 & 0 & 0 & 1\\ 0 & a_{4} & a_{5} & a_{6} \end{array}\right]=\left[\begin{array}{cccc} 0 & 1 & 0 & 0\\ 0 & \frac{K_{f}+K_{r}}{mv_{x}} & -\frac{K_{f}+K_{r}}{m} & \frac{aK_{f}-bK_{r}}{mv_{x}}\\ 0 & 0 & 0 & 1\\ 0 & \frac{{aK}_{f}-bK_{r}}{I_{z}v_{x}} & -\frac{aK_{f}-bK_{r}}{I_{z}} & \frac{{a^{2}K}_{f}-b^{2}K_{r}}{I_{z}v_{x}} \end{array}\right];\;B=\left[\begin{array}{c} 0\\ b_{1}\\ 0\\ b_{2} \end{array}\right]=\left[\begin{array}{c} 0\\ -\frac{K_{f}}{m}\\ 0\\ -\frac{{aK}_{r}}{I_{z}} \end{array}\right];$$

$$C=\left[\begin{array}{cc} 0 & 0\\ 1 & 0\\ 0 & 0\\ 0 & 1 \end{array}\right];\;\omega =\left[\begin{array}{c} f_{1}\\ f_{2} \end{array}\right]=\left[\begin{array}{c} \left(\frac{{aK}_{f}-bK_{r}}{mv_{x}}-v_{x}\right){\dot{\psi }}_{r}\\ \frac{{a^{2}K}_{f}+b^{2}K_{r}}{I_{z}v_{x}}{\dot{\psi }}_{r} \end{array}\right].$$

## 3. Realization of Generalized Hamilton in Vehicle Systems and Controller Design

Due to the simplicity of the structure of the vehicle-dynamics control system, the realization of the feedback-dissipative Hamilton form is straightforward. Furthermore, in the following section, which discusses the design of the GHRC for this system, it is also necessary to have the feedback-dissipative Hamilton realization of the intelligent vehicle trajectory-tracking control system. Therefore, this section first provides a detailed derivation, explanation and validation of the feedback-dissipative Hamilton realization of the intelligent vehicle trajectory-tracking control system. Next, a brief explanation of the feedback-dissipative Hamilton realization of the vehicle-dynamics control system is given.

### 3.1. Intelligent Vehicle Trajectory Tracking Control System

#### 3.1.1. Orthogonal Decomposition Hamilton Realization

In this paper, the system Hamilton realization of vehicle systems is carried out using the orthogonal decomposition method. First, the vehicle system Equation (8) is rewritten in the following form:

(9) $$\dot{\xi }=f\left(\xi \right)+g_{1}\left(\xi \right)\mu +g_{2}(\xi )\omega$$

Let

$$f\left(\xi \right)=A\xi =\left[\begin{array}{c} \xi_{2}\\ a_{1}\xi_{2}+a_{2}\xi_{3}+a_{3}\xi_{4}\\ \xi_{4}\\ a_{4}\xi +a_{5}\xi_{3}+a_{6}\xi_{4} \end{array}\right];\;g_{1}\left(\xi \right)=B=\left[\begin{array}{c} 0\\ b_{1}\\ 0\\ b_{2} \end{array}\right];\;g_{2}\left(\xi \right)=C=\left[\begin{array}{cc} 0 & 0\\ 1 & 0\\ 0 & 0\\ 0 & 1 \end{array}\right].$$

The selection of Hamilton Function is as follows:

(10) $$H\left(\xi \right)=\frac{1}{2}(\xi_{1}^{2}+\xi_{2}^{2}+\xi_{3}^{2}+\xi_{4}^{2})$$

Its partial derivatives are represented as:

(11) $$\nabla H={\left[\begin{array}{cccc} \xi_{1} & \xi_{2} & \xi_{3} & \xi_{4} \end{array}\right]}^{T}$$

To make the equipotential surface of H(ξ), at any point ξ≠0, decompose f(ξ) into the direction of the gradient vector ∇H and tangent plane.

(12) $$f\left(\xi \right)=f_{gd}\left(\xi \right)+f_{td}(\xi )$$

Let $\varsigma =\xi_{1}^{2}+\xi_{2}^{2}+\xi_{3}^{2}+\xi_{4}^{2}$, then in Equation (12):

(13) $$f_{gd}\left(\xi \right)=\frac{\left\langle f\right.,\left.\nabla H\right\rangle }{{\left\|H\right\|}^{2}}\nabla H={\frac{1}{\varsigma }\left[\begin{array}{cccc} \tau_{1} & \tau_{2} & \tau_{3} & \tau_{4} \end{array}\right]}^{T}=\frac{1}{\varsigma }\left[\begin{array}{c} \xi_{1}{(\xi }_{2}\left(a_{1}\xi_{2}+a_{2}\xi_{3}+a_{3}\xi_{4}\right)+\xi_{4}\left(a_{4}\xi_{2}+a_{5}\xi_{3}+a_{6}\xi_{4}\right)+\xi_{1}\xi_{2}+\xi_{3}\xi_{4})\\ {\xi_{2}(\xi }_{2}\left(a_{1}\xi_{2}+a_{2}\xi_{3}+a_{3}\xi_{4}\right)+\xi_{4}\left(a_{4}\xi_{2}+a_{5}\xi_{3}+a_{6}\xi_{4}\right)+\xi_{1}\xi_{2}+\xi_{3}\xi_{4})\\ {\xi_{3}(\xi }_{2}\left(a_{1}\xi_{2}+a_{2}\xi_{3}+a_{3}\xi_{4}\right)+\xi_{4}\left(a_{4}\xi_{2}+a_{5}\xi_{3}+a_{6}\xi_{4}\right)+\xi_{1}\xi_{2}+\xi_{3}\xi_{4})\\ {\xi_{4}(\xi }_{2}\left(a_{1}\xi_{2}+a_{2}\xi_{3}+a_{3}\xi_{4}\right)+\xi_{4}\left(a_{4}\xi_{2}+a_{5}\xi_{3}+a_{6}\xi_{4}\right)+\xi_{1}\xi_{2}+\xi_{3}\xi_{4}) \end{array}\right]$$

(14) $$f_{td}\left(\xi \right)=f\left(\xi \right)-f_{gd}\left(\xi \right)={\left[\begin{array}{cccc} \vartheta_{1} & \vartheta_{2} & \vartheta_{3} & \vartheta_{4} \end{array}\right]}^{T}=\frac{1}{\varsigma }\left[\begin{array}{c} \xi_{2}\varsigma -\tau_{1}\\ {(a}_{1}\xi_{2}+a_{2}\xi_{3}+a_{3}\xi_{4})\varsigma -\tau_{2}\\ x_{4}\varsigma -\tau_{3}\\ \left(a_{4}\xi_{2}+a_{5}\xi_{3}+a_{6}\xi_{4}\right)\varsigma -\tau_{4} \end{array}\right]$$

Thus,

(15) $$J\left(\xi \right)=\frac{1}{{\left\|H\right\|}^{2}}\left[f_{td}\left(\xi \right)\nabla H^{T}-\nabla Hf_{td}^{T}\left(\xi \right)\right]=\frac{1}{\varsigma }\left[\begin{array}{cccc} 0 & \vartheta_{1}\xi_{2}-\vartheta_{2}\xi_{1} & \vartheta_{1}\xi_{3}-\vartheta_{3}\xi_{1} & \vartheta_{1}\xi_{4}-\vartheta_{4}\xi_{1}\\ \vartheta_{2}\xi_{1}-\vartheta_{1}\xi_{2} & 0 & \vartheta_{2}\xi_{3}-\vartheta_{3}\xi_{2} & \vartheta_{2}\xi_{4}-\vartheta_{4}\xi_{2}\\ \vartheta_{3}\xi_{1}-\vartheta_{1}\xi_{3} & \vartheta_{3}\xi_{2}-\vartheta_{2}\xi_{3} & 0 & \vartheta_{3}\xi_{4}-\vartheta_{4}\xi_{3}\\ \vartheta_{4}\xi_{1}-\vartheta_{1}\xi_{4} & \vartheta_{4}\xi_{2}-\vartheta_{2}\xi_{4} & \vartheta_{4}\xi_{3}-\vartheta_{3}\xi_{4} & 0 \end{array}\right]$$

(16) $$S\left(\xi \right)=\frac{\left\langle f\right.,\left.\nabla H\right\rangle }{{\left\|H\right\|}^{2}}I_{4}=\frac{1}{\varsigma }\left[\begin{array}{cccc} \kappa_{s} & 0 & 0 & 0\\ 0 & \kappa_{s} & 0 & 0\\ 0 & 0 & \kappa_{s} & 0\\ 0 & 0 & 0 & \kappa_{s} \end{array}\right]$$

where, $\kappa_{s}=\xi_{2}\left(a_{1}\xi_{2}+a_{2}\xi_{3}+a_{3}\xi_{4}\right)+\xi_{4}\left(a_{4}\xi_{2}+a_{5}\xi_{3}+a_{6}\xi_{4}\right)+\xi_{1}\xi_{2}+\xi_{3}\xi_{4}$ and $I_{4}$ is a 4×4 identity matrix.

When ξ≠0, then:

(17) $$\dot{\xi }=\left[J\left(\xi \right)+S\left(\xi \right)\right]\nabla H+g_{1}\left(\xi \right)\mu +g_{2}(\xi )\omega$$

Therefore, the system given by Equation (9) can be implemented as a Hamilton system:

(18) $$\dot{\xi }=\left\{\begin{array}{l} \left[J\left(\xi \right)+S\left(\xi \right)\right]\nabla H+g_{1}\left(\xi \right)\mu +g_{2}\left(\xi \right)\omega \;\;\;\;,\xi \neq 0\\ g_{1}\left(\xi \right)\mu +g_{2}\left(\xi \right)\omega \;\;\;\;\;\;\;\;\;\;\;\;\;\;\;\;\;\;\;\;\;\;\;\;\;\;\;\;\;\;\;\;\;\;\;\;\;\;\;,\xi =0 \end{array}\right.$$

#### 3.1.2. Hamilton Dissipative Controller Design

In, $R^{4}$ consider the equation $L_{g1}H=0$, denoted by ${Ʃ}_{1}$ represented as a hypersurface. Let $\xi \in R^{4}$ be a given point. If$\;\xi \notin {Ʃ}_{1}$, then $L_{g1}H\neq 0$. In this case, we decompose S(ξ) as follows:

(19) $$S\left(\xi \right)=-P_{1}\left(\xi \right)+P_{2}(\xi )$$

Select

(20) $$P_{1}\left(\xi \right)=\frac{1}{\varsigma }{\iota_{2}I}_{4}$$

(21) $$P_{2}\left(\xi \right)=\frac{1}{\varsigma }{\iota_{1}I}_{4}$$

where $\iota_{1}=\left(a_{1}+2.3\right)\xi_{2}^{2}+\xi_{1}\xi_{2}+a_{2}\xi_{2}\xi_{3}+a_{3}\xi_{2}\xi_{4}+\xi_{3}\xi_{4}+a_{4}\xi_{2}\xi_{4}+a_{5}\xi_{3}\xi_{4}+{(a}_{6}+2.3)\xi_{4}^{2}$; $\iota_{2}=2.3\times (\xi_{2}^{2}+\xi_{4}^{2})$; $P_{1}>0$; $P_{2}$ symmetry.

The trajectory-tracking Hamilton dissipative controller (TTHDC) is taken:

(22) $$\mu_{1}=\left\{\begin{array}{l} \frac{1}{L_{g1}H}\left[L_{g1}Hv-\nabla H^{T}P_{2}\left(\xi \right)\nabla H\right]\;\;\;\;,\xi \neq 0\\ 0\;\;\;\;\;\;\;\;\;\;\;\;\;\;\;\;\;\;\;\;\;\;\;\;\;\;\;\;\;\;\;\;\;\;\;\;\;\;\;\;\;\;\;\;\;\;\;\;\;\;\;\;\;\;,\xi =0 \end{array}\right.=\left\{\begin{array}{l} v-\frac{\iota_{1}\varsigma }{\varsigma (b_{1}\xi_{2}+b_{2}\xi_{4})}\;\;\;\;,\xi \neq 0\\ 0\;\;\;\;\;\;\;\;\;\;\;\;\;\;\;\;\;\;\;\;\;\;\;\;\;\;\;\;,\xi =0 \end{array}\right.$$

where ν is a newly introduced control parameter; ν=0 when only TTHDC acts.

Applying the controller in Equation (22) to Equation (18), we obtain:

(23) $$\dot{\xi }=\left\{\begin{array}{l} \left[J\left(\xi \right)+\tilde{J}\left(\xi \right)-P_{1}(\xi )\right]\nabla H+g_{1}\left(\xi \right)v+g_{2}\left(\xi \right)\omega \;\;\;\;,\xi \neq 0\\ g_{2}\left(\xi \right)\omega \;\;\;\;\;\;\;\;\;\;\;\;\;\;\;\;\;\;\;\;\;\;\;\;\;\;\;\;\;\;\;\;\;\;\;\;\;\;\;\;\;\;\;\;\;\;\;\;\;\;\;\;\;\;\;\;\;\;\;\;\;\;\;\;\;\;\;\;\;\;\;,\xi =0 \end{array}\right.$$

where

(24) $$\tilde{J}\left(\xi \right)=\frac{1}{L_{g1}H}\left[P_{2}\left(\xi \right)\nabla Hg_{1}^{T}-g_{1}\nabla H^{T}P_{2}\left(\xi \right)\right]=\frac{1}{\varsigma (b_{1}\xi_{2}+b_{2}\xi_{4})}\left[\begin{array}{cccc} 0 & b_{1}\iota_{1}\xi_{1} & 0 & b_{2}\iota_{1}\xi_{1}\\ -b_{1}\iota_{1}\xi_{1} & 0 & -b_{1}\iota_{1}\xi_{3} & b_{2}\iota_{1}\xi_{2}-b_{1}\iota_{1}\xi_{4}\\ 0 & b_{1}\iota_{1}\xi_{3} & 0 & b_{2}\iota_{1}\xi_{3}\\ -b_{2}\iota_{1}\xi_{1} & {-b}_{2}\iota_{1}\iota_{2}+b_{1}\iota_{1}\xi_{4} & -b_{2}\iota_{1}\xi_{3} & 0 \end{array}\right]$$

As J(ξ) is antisymmetric and $P_{1}>0$, the system in Equation (24) is strictly dissipative.

Since

(25) $$L_{g1}H=g_{1}^{T}\nabla H=b_{1}\xi_{2}+b_{2}\xi_{4}\neq 0,$$

Equation (18) has a strict feedback-dissipative Hamilton realization.

Therefore, the feedback-dissipative Hamilton realization of the intelligent-vehicle- trajectory-tracking control system can be achieved through the state feedback controller $\mu_{1}$, and the dissipative form is given by Equation (24). In addition, since the Hamilton function H(ξ) is Lyapunov function of the system and remains invariant during the control processes, the system Equation (18) will eventually converge to the origin (v=0).

In connection with the design of the vehicle dissipation system in this section, and then from literature [24] and literature [25], the storage function $H\left(\xi \right)$($\nabla H{|}_{\xi =0}=0,\nabla H{|}_{\xi \neq 0}\neq 0$) and the supply rate s(ξ,y) should satisfy the differential dissipation inequality.

(26) $$\nabla H^{T}[f\left(\xi \right)+g_{1}\left(\xi \right)\mu_{1}]\leq s\left(\xi ,y\right)$$

where the supply rate $s\left(\xi ,y\right)=\mu_{1}^{T}y$ and the system output $y=g_{1}^{T}(\xi )\nabla H$.

According to the selection of the storage function $H\left(\xi \right)$ in Equation (10) and the design of the control rate $\mu_{1}$ in Equation (22), substituting it into Equation (26) shows that if the differential dissipation inequality Equation (26) holds, the parameter $\kappa_{s}$ should not be greater than 0; that is, the function $S\left(\xi \right)\leq 0$. Again, since $\dot{\xi }=f(\xi )$ is asymptotically stable, having $\dot{H}=\frac{\partial H^{T}}{\partial \xi }\dot{\xi }=L_{f}H<0$ for the Lyapunov function $H\left(\xi \right)$ makes $S\left(\xi \right)<0$, holding in the orthogonal decomposition realization of Equation (16), which makes the differential dissipation inequality Equation (26) hold.

### 3.2. Intelligent Vehicle Dynamic Control System

From a given reference trajectory $\left(y_{r}\;\psi_{r}\right)$ corresponding to its derivative as $\left({\dot{y}}_{r}{\dot{\psi }}_{r}\right)$, when $\delta_{f}=0$ is given in Equation (6), the system equation ${\dot{\xi }}_{r}^{\prime }=A^{\prime }\xi_{r}^{\prime }$ is obtained by substituting the reference value $\left({\dot{y}}_{r}\;{\dot{\psi }}_{r}\right)$ into Equation (6). According to the two degree of freedom vehicle-dynamical system model, the control objective of the vehicle-trajectory tracking is to make the deviation of the current vehicle from the desired trajectory as close to 0 as possible through the design of the appropriate Hamilton dissipative controller.

In accordance with the above control objectives and in conjunction with the difference between the vehicle system Equation (6) and the system equation ${\dot{\xi }}_{r}^{\prime }=A^{\prime }\xi_{r}^{\prime }$, Equation (6) can be rewritten as follows:

(27) $${\dot{\xi }}^{\prime }=f_{1}\left(\xi^{\prime }\right)+g_{1}^{\prime }\left(\xi^{\prime }\right)\mu^{\prime }$$

In this equation:

$$\xi^{\prime }=\left[\begin{array}{c} \xi_{1}^{\prime }\\ \xi_{2}^{\prime } \end{array}\right]=\left[\begin{array}{c} \dot{y}-{\dot{y}}_{r}\\ \dot{\psi }-{\dot{\psi }}_{r} \end{array}\right];\;f_{1}\left(\xi^{\prime }\right)=A^{\prime }\xi^{\prime }=\left[\begin{array}{cc} a_{11} & a_{12}\\ a_{21} & a_{22} \end{array}\right]\xi^{\prime }=\left[\begin{array}{cc} \frac{K_{f}+K_{r}}{mv_{x}} & \frac{{aK}_{f}-bK_{r}}{mv_{x}}-v_{x}\\ \frac{{aK}_{f}-bK_{r}}{I_{z}v_{x}} & \frac{{a^{2}K}_{f}+b^{2}K_{r}}{I_{z}v_{x}} \end{array}\right]\xi^{\prime };\;\mu^{\prime }=\delta_{f};\;g_{1}^{\prime }\left(\xi^{\prime }\right)=\left[\begin{array}{c} b_{1}^{\prime }\\ b_{2}^{\prime } \end{array}\right]=\left[\begin{array}{c} -\frac{K_{f}}{m}\\ -\frac{{aK}_{r}}{I_{z}} \end{array}\right].$$

According to the two degree of freedom vehicle-dynamics error model (27), the Hamilton function is selected so that the tracking error converges to zero:

(28) $$H^{\prime }(\xi^{\prime })=\frac{1}{2}\xi_{1}^{\prime 2}+\frac{1}{2}\xi_{2}^{\prime 2}$$

Its partial derivatives are represented as:

(29) $$\nabla H^{\prime }={\left[\begin{array}{cc} \xi_{1}^{\prime } & \xi_{2}^{\prime } \end{array}\right]}^{T}$$

By following the calculation steps based on Equations (12)–(16), we can obtain:

(30) $$J^{\prime }\left(\xi^{\prime }\right)=\frac{1}{\xi_{1}^{\prime 2}+\xi_{2}^{\prime 2}}\left[\begin{array}{cc} 0 & J_{12}^{\prime }\\ -J_{12}^{\prime } & 0 \end{array}\right]$$

(31) $$S^{\prime }\left(\xi^{\prime }\right)=\frac{1}{\xi_{1}^{\prime 2}+\xi_{2}^{\prime 2}}\left[\begin{array}{cc} S_{11}^{\prime } & 0\\ 0 & S_{11}^{\prime } \end{array}\right]$$

In these two equations:

$J_{12}^{\prime }=a_{11}{(\xi }_{1}^{\prime 3}\xi_{2}^{\prime }+\xi_{1}^{\prime }\xi_{2}^{\prime 3})+a_{12}{(\xi }_{1}^{\prime 2}\xi_{2}^{\prime 2}+\xi_{2}^{\prime 4})-a_{21}\left(\xi_{1}^{\prime 4}+\xi_{1}^{\prime 2}\xi_{2}^{\prime 2}\right)-a_{22}{(\xi }_{1}^{\prime 3}\xi_{2}^{\prime }+\xi_{1}^{\prime }\xi_{2}^{\prime 3})$;
$S_{11}^{\prime }=a_{11}\xi_{1}^{\prime 2}+a_{12}\xi_{1}^{\prime }\xi_{2}^{\prime }+a_{21}\xi_{1}^{\prime }\xi_{2}^{\prime }+a_{22}\xi_{2}^{\prime 2}$.

When $\xi^{\prime }\neq 0$, we decompose $S^{\prime }(\xi^{\prime })$ as follows:

(32) $$S^{\prime }\left(\xi^{\prime }\right)=-P_{1}^{\prime }\left(\xi^{\prime }\right)+P_{2}^{\prime }\left(\xi^{\prime }\right)$$

Select,

(33) $$P_{1}^{\prime }\left(\xi^{\prime }\right)=\frac{1}{\xi_{1}^{\prime 2}+\xi_{2}^{\prime 2}}\left[\begin{array}{cc} \iota_{2}^{\prime } & 0\\ 0 & \iota_{2}^{\prime } \end{array}\right]$$

(34) $$P_{2}^{\prime }\left(\xi^{\prime }\right)=\frac{1}{\xi_{1}^{\prime 2}+\xi_{2}^{\prime 2}}\left[\begin{array}{cc} \iota_{1}^{\prime } & 0\\ 0 & \iota_{1}^{\prime } \end{array}\right]$$

where $\iota_{1}^{\prime }={(a}_{12}+a_{21}{)\xi }_{1}^{\prime }\xi_{2}^{\prime }$; $\iota_{2}^{\prime }=\xi_{1}^{\prime 2}+\xi_{2}^{\prime 2}$; $P_{1}^{\prime }>0$; $P_{2}^{\prime }$ symmetry.

The dynamics Hamilton dissipative controller (DHDC) is taken:

(35) $$\mu_{1}^{\prime }=\left\{\begin{array}{l} v^{\prime }-\frac{\iota_{1}^{\prime }\left(\xi_{1}^{\prime 2}+\xi_{2}^{\prime 2}\right)}{\left(\xi_{1}^{\prime 2}+\xi_{2}^{\prime 2}\right)\left(b_{1}^{\prime }\xi_{1}^{\prime }+b_{2}^{\prime }\xi_{2}^{\prime }\right)}\;\;\;\;,\xi^{\prime }\neq 0\\ 0\;\;\;\;\;\;\;\;\;\;\;\;\;\;\;\;\;\;\;\;\;\;\;\;\;\;\;\;\;\;\;\;\;\;\;\;\;\;\;\;\;,\xi^{\prime }=0 \end{array}\right.$$

where $\nu^{\prime }$ is a newly introduced control parameter, $\nu^{\prime }=0\;$when only DHDC acts.

Based on Equation (35) and in conjunction with the form of system Equation (22), we obtain:

(36) $${\dot{\xi }}^{\prime }=\left\{\begin{array}{l} \left[J^{\prime }\left(\xi^{\prime }\right)+{\tilde{J}}^{\prime }\left(\xi^{\prime }\right)-P_{1}^{\prime }(\xi^{\prime })\right]\nabla H^{\prime }+g_{1}^{\prime }\left(\xi^{\prime }\right)v^{\prime }\;\;\;,\xi^{\prime }\neq 0\\ 0\;\;\;\;\;\;\;\;\;\;\;\;\;\;\;\;\;\;\;\;\;\;\;\;\;\;\;\;\;\;\;\;\;\;\;\;\;\;\;\;\;\;\;\;\;\;\;\;\;\;\;\;\;\;\;\;\;\;\;\;\;\;\;\;\;\;\;\;\;\;\;\;\;\;\;\;,\xi^{\prime }=0 \end{array}\right.$$

where

(37) $$J^{\prime }(\xi^{\prime })=\frac{1}{\left(\xi_{1}^{\prime 2}+\xi_{2}^{\prime 2}\right)\left(b_{1}^{\prime }\xi_{1}^{\prime }+b_{2}^{\prime }\xi_{2}^{\prime }\right)}\left[\begin{array}{cc} 0 & \iota_{1}^{\prime }b_{2}^{\prime }\xi_{1}^{\prime }-\iota_{1}^{\prime }b_{1}^{\prime }\xi_{2}^{\prime }\\ \iota_{1}^{\prime }b_{1}^{\prime }\xi_{2}^{\prime }-\iota_{1}^{\prime }b_{2}^{\prime }\xi_{1}^{\prime } & 0 \end{array}\right]$$

As $J^{\prime }(\xi^{\prime })$ is antisymmetric and $P_{1}^{\prime }>0$, the system in Equation (36) is strictly dissipative.

Similar to the above analysis of the dissipation inequality Equation (25) in the intelligent vehicle-trajectory tracking control system, according to the selection of the storage function $H^{\prime }\left(\xi^{\prime }\right)$ in Equation (28) and the design of the control rate $\mu_{1}^{\prime }$ in Equation (35), substituting it into Equation (26) shows that, if Equation (26) is made to be valid, the parameter $S_{11}^{\prime }$ should not be greater than 0, i.e., the function $S^{\prime }\left(\xi^{\prime }\right)\leq 0$. Again, since ${\dot{\xi }}^{\prime }=f_{1}(\xi^{\prime })$ is asymptotically stable at the equilibrium point, for the Lyapunov function $H^{\prime }\left(\xi^{\prime }\right)$ there is $\dot{H^{\prime }}=\frac{\partial H^{\prime T}}{\partial \xi^{\prime }}{\dot{\xi }}^{\prime }=L_{f_{1}}H^{\prime }<0$, which then makes it possible to realize in the orthogonal decomposition that Equation (31) holds for $S^{\prime }\left(\xi^{\prime }\right)<0$, which in turn makes Equation (26) hold for the differential dissipation inequality.

## 4. Robust Controller Design for Dissipative Hamilton System of Intelligent Vehicles

According to the aforementioned realization of a dissipative Hamilton for intelligent vehicle systems and the design of dissipative Hamilton controllers, as well as the analysis of the simulation results in Section 5, it is imperative to further develop a robust Hamilton controller to improve the accuracy and stability of vehicle-trajectory tracking control. Like the previous two controllers, based on the predetermined reference trajectory and the output of the Carsim vehicle model, the necessary state variables and their errors are obtained. An appropriate Hamilton robust controller is then designed to minimize the derivatives of the lateral and heading errors throughout the trajectory tracking processes. The design generates the control input of the FWSA required by the vehicle systems and finally achieves a good trajectory tracking control performance.

To investigate the robust control of dissipative Hamilton system for intelligent vehicle-trajectory tracking, the following lemma is presented first [26].

**Lemma 1.**  *Consider an intelligent vehicle trajectory-tracking control system,*

(38) $$\left\{\begin{array}{l} \dot{\xi }=f\left(\xi \right)+g_{1}\left(\xi \right)\mu +g_{2}\left(\xi \right)\omega ,f\left(\xi_{0}\right)=0\\ z=h(\xi )\;\;\;\;\;\;\;\; \end{array}\right.$$

*where $\xi \in R^{n}$ represents the system state, $\omega \in R^{s}$ represents the disturbance and $z\in R^{q}$ represents the evaluation signal.*

*If there exists a function* $V(\xi )\geq 0\;(V(\xi_{0})\geq 0)$*, such that the Hamilton–Jacobi inequality:*

(39) $${\left(\frac{\partial V}{\partial \xi }\right)}^{T}f_{1}\left(\xi \right)+\frac{1}{2\lambda^{2}}{\left(\frac{\partial V}{\partial \xi }\right)}^{T}g_{2}g_{2}^{T}\frac{\partial V}{\partial \xi }+\frac{1}{2}h^{T}h\leq 0$$

*holds, then the* $L_{2}$ *gain (from* ω *to* z*) of system (39) is no more than* λ*, i.e.,*

(40) $$\int_{0}^{T}{\left\|z\left(t\right)\right\|}^{2}dt\leq \lambda^{2}\int_{0}^{T}{\left\|\omega \left(t\right)\right\|}^{2}dt,\forall \omega \in L_{2}[0,T]$$

*where* λ>0.

We now investigate the robust control of the dissipative Hamilton system for intelligent vehicle-trajectory tracking proposed in this paper. Based on Equation (23), the following dissipative Hamilton system is obtained:

(41) $$\left\{\begin{array}{l} \dot{\xi }=\left[\bar{J}\left(\xi \right)-P_{1}(\xi )\right]\nabla H+g_{1}\left(\xi \right)\mu_{2}+g_{2}\left(\xi \right)\omega \\ {y=g}_{2}^{T}\left(\xi \right)\nabla H\;\;\;\;\;\;\;\;\;\\ {z=r\left(\xi \right)g}_{1}^{T}\left(\xi \right)\nabla H\;\;\;\;\;\;\;\;\;\;\;\;\;\;\;\;\;\;\;\;\;\;\;\;\;\;\;\;\;\;\;\;\;\;\;\;\;\;\;\;\;\;\;\;\;\;\; \end{array}\right.$$

In this equation, $\xi \in R^{4}$; $\mu_{2}=\nu$ and $\mu_{2}\in R$; $\omega \in R^{2}$ represents the disturbance; $y\in R^{2}$ is the output; z∈R is the evaluation signal; r(ξ) is a full rank weight matrix; $\bar{J}\left(\xi \right)=J\left(\xi \right)+\tilde{J}(\xi )$ is skew symmetric; $P_{1}(\xi )\geq 0$ is a positive semi-definite matrix. It is assumed that H(ξ) reaches a local minimum at the equilibrium point $\xi_{0}$.

The H∞ control design problem for the dissipative Hamilton system (41) is to find a suitable state feedback control rate $\mu_{2}=\alpha (\xi )\;(\alpha (\xi_{0})=0)$ for a given suppression level λ>0, such that the $L_{2}$ gain (from ω to z) of the closed-loop system is no greater than λ, i.e., Equation (40) holds and the closed-loop system is asymptotically stable when ω=0.

To design the H∞ controller, the following two assumptions are made:

1. $H\left(\xi \right)\in C^{2}$, and Hessian matrix $Hess\left(H\left(\xi_{0}\right)\right)>0$;
2. $r\left(\xi \right)g_{1}^{T}\nabla H=0,\;\forall \xi \in R^{n}.$

In assumption 1, $H\left(\xi \right)\in C^{2}$ ensures the existence of Hess(H(ξ)) and $Hess(H(\xi_{0}))>0$ guarantees that H(ξ) is strictly concave in a neighborhood of the equilibrium point $\xi_{0}$. Assumption 2 is known as the orthogonality condition, which is a common assumption in H∞ control [27].

**Theorem 1.**  *Assuming 1 and 2 hold, and satisfy:*

(42) $$P_{1}\left(\xi \right)+\frac{1}{2\lambda^{2}}(g_{1}g_{1}^{T}-g_{2}g_{2}^{T})\geq 0,$$

*the* H∞ *control problem for system (41) can be solved by the following control rate:*

(43) $$\mu_{2}=-\left(\frac{1}{2}r^{T}\left(\xi \right)r\left(\xi \right)+\frac{1}{2\lambda^{2}}I_{m}\right)g_{1}^{T}\left(\xi \right)\nabla H=-\frac{r^{2}\lambda^{2}+1}{2\lambda^{2}}(b_{1}\xi_{2}+b_{2}\xi_{4})$$

*In the equation,* λ *represents the disturbance attenuation level, and* r(ξ) *is a full rank weight matrix.*

**Proof.**  Substituting Equation (43) into the system Equation (41) yields:

(44) $$\left\{\begin{array}{l} \dot{\xi }=\left[\bar{J}\left(\xi \right)-{(P}_{1}\left(\xi \right)+\frac{1}{2}g_{1}r^{T}rg_{1}^{T}+\frac{1}{2\lambda^{2}}g_{1}g_{1}^{T})\right]\nabla H+g_{2}\left(\xi \right)\omega ≔f_{1}\left(\xi \right)+g_{2}\left(\xi \right)\omega \\ {y=g}_{2}^{T}\nabla H\;\;\;\;\;\;\;\;\;\;\;\;\;\;\\ {z=rg}_{1}^{T}\nabla H≔h\left(\xi \right)\;\;\;\;\;\;\;\;\; \end{array}\right.$$

To verify the stability of the dissipative Hamilton H∞ controller for trajectory tracking of intelligent driving vehicles, we choose the Lyapunov function V(ξ)=H(ξ)≥0. Combining Equation (42), Equation (44) and assumption 2, the Hamilton–Jacobi inequality of the system can be expressed as follows:

(45) $$\begin{array}{l} {\left(\frac{\partial V}{\partial \xi }\right)}^{T}f_{1}\left(\xi \right)+\frac{1}{2\lambda^{2}}{\left(\frac{\partial V}{\partial \xi }\right)}^{T}g_{2}g_{2}^{T}\frac{\partial V}{\partial \xi }+\frac{1}{2}h^{T}h\\ =-\nabla H^{T}{(P}_{1}\left(\xi \right)+\frac{1}{2}g_{1}r^{T}rg_{1}^{T}+\frac{1}{2\lambda^{2}}g_{1}g_{1}^{T})\nabla H+\frac{1}{2\lambda^{2}}\nabla H^{T}g_{2}g_{2}^{T}\nabla H+\frac{1}{2}\nabla H^{T}g_{1}r^{T}rg_{1}^{T}\nabla H\\ =-\nabla H^{T}{(P}_{1}\left(\xi \right)+\frac{1}{2\lambda^{2}}(g_{1}g_{1}^{T}-g_{2}g_{2}^{T}))\nabla H\leq 0 \end{array}$$

According to Lemma 1, the $L_{2}$ gain (from ω to z) of the system Equation (44) is not greater than λ.

Next, we will prove that the system Equation (44) is asymptotically stable (ω=0). When ω=0, it can be obtained from Equations (42) and (44):

(46) $$\begin{array}{l} {\dot{V}\left(\xi \right)=\left(\frac{\partial V}{\partial \xi }\right)}^{T}\left[\bar{J}\left(\xi \right)-{(P}_{1}\left(\xi \right)+\frac{1}{2}g_{1}r^{T}rg_{1}^{T}+\frac{1}{2\lambda^{2}}g_{1}g_{1}^{T})\right]\nabla H\\ \;\;=-\nabla H^{T}{[P}_{1}\left(\xi \right)+\frac{1}{2\lambda^{2}}\left(g_{1}g_{1}^{T}-g_{2}g_{2}^{T}\right)]\nabla H-\frac{1}{2\lambda^{2}}\nabla H^{T}g_{2}g_{2}^{T}\nabla H-\frac{1}{2}\nabla H^{T}g_{1}r^{T}rg_{1}^{T}\nabla H\\ \;\;\leq -\frac{1}{2\lambda^{2}}\nabla H^{T}g_{2}g_{2}^{T}\nabla H-\frac{1}{2}\nabla H^{T}g_{1}r^{T}rg_{1}^{T}\nabla H\;\;\;\\ \;\;=-\frac{1}{2\lambda^{2}}(\xi_{2}^{2}+\xi_{4}^{2})-\frac{r^{2}}{2}{\left(b_{1}\xi_{2}+b_{2}\xi_{4}\right)}^{2}\leq 0 \end{array}$$

Therefore, it can be seen that the closed-loop system converges to the largest invariant set contained in the following set.

(47) $$\left\{\xi :\dot{V}\left(\xi \right)=0\right\}\subset \left\{\xi :\xi_{2}^{2}+\xi_{4}^{2}\equiv 0,b_{1}\xi_{2}+b_{2}\xi_{4}\equiv 0,\forall t\geq 0\right\}≔S$$

From Equation (46), it can be observed that when $\dot{V}=0$, $\xi_{2}=\xi_{4}=0,\forall t\;\dot{\xi_{2}}=\dot{\xi_{4}}=0$. Combining this observation with the lateral error calculation formula in Equation (7) and the control system for intelligent vehicle-trajectory tracking in Equation (8), it can be concluded that $\xi_{1}=\xi_{3}=0$. According to LaSalle’s invariance principle [28], the closed-loop system described by Equation (44) can be concluded when ω = 0, which is asymptotically stable. Therefore, the H∞ control problem for the dissipative Hamilton system (41) of intelligent vehicle-trajectory tracking can be achieved by the control rate given by Equation (43). □

## 5. Simulation Analysis

Using the simulation platform built using Carsim and MATLAB/Simulink, taking the double lane-change and lane-change trajectories as the reference trajectories, the road surface friction coefficient was set to 0.85, and the trajectory-tracking control effect of each controller on the vehicle was verified via simulation at three speeds: 36 km/h, 54 km/h and 72 km/h, respectively. First, the simulation results of the two aforementioned Hamilton dissipative controllers designed based on different vehicle system models were presented and compared. Next, in order to better illustrate the superiority of the Hamilton robust control method in the trajectory-tracking accuracy and stability of intelligent vehicles, simulations and comparisons were performed with two common trajectory-tracking control algorithms, SMC [29] and LQR [30], as benchmarks. Table 1 shows some parameters of the vehicle and its GHRC.

A large amount of simulation data was obtained by changing the values of r (GHRC weighting coefficients) and λ (GHRC suppression level) when selecting the controller parameters. Furthermore, based on the observation of the trajectory tracking effect and the calculation of the optimization results, we found that when r>0.05 and λ<8, the lateral error of the vehicle-trajectory tracking will be larger and the vehicle-trajectory tracking effect will be slightly worse. On the contrary, the lateral error will be smaller and the trajectory-tracking effect will be slightly better, but it will make the control stability of the controller in the middle and high speeds become poor. Therefore, after comprehensive consideration of various aspects, the parameters of GHRC are finally selected as r=0.05, λ=8.

### 5.1. Simulation Scenarios

**Figure 3:**
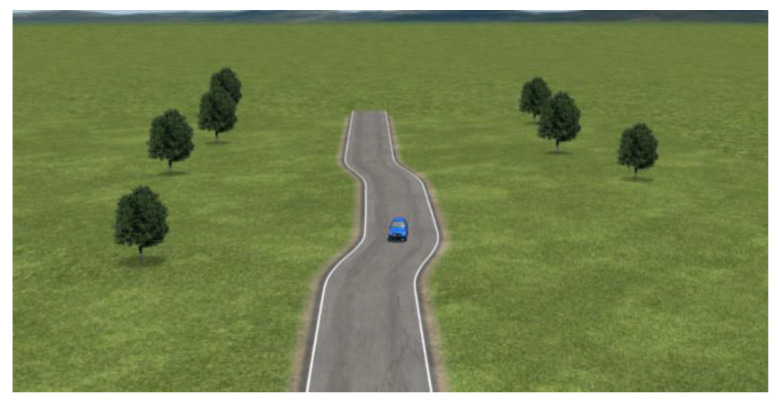
Schematic diagram of the Carsim double-lane road.

The standard double lane-change maneuver, as shown in Figure 3, is a closed-loop testing condition that is frequently used in practical automotive stability testing and closed-loop simulation experiments with driver models.

The expressions for the desired lateral position $y_{r}$ and desired yaw angle $\psi_{r}$ of the double lane-change maneuver used in this paper are as follows [31]:

(48) $$y_{r}=\left(\frac{dn_{1}}{2}\left(1+\tanh\left(z_{1}\right)\right)-\frac{dn_{2}}{2}\left(1+\tanh\left(z_{2}\right)\right)\right)$$

(49) $$\psi_{r}=\mathrm{atan}\left(\frac{1.2dn_{1}}{dm_{1}}{\left(\frac{1}{\cos\left(z_{1}\right)}\right)}^{2}-\frac{1.2dn_{2}}{dm_{2}}{\left(\frac{1}{\cos\left(z_{2}\right)}\right)}^{2}\right)$$

where $z_{1}=0.095\times (x_{0}-60)-1.2$; $z_{2}=0.095\times (x_{0}-120)-1.2$; $dm_{1}=25$; ${dm}_{2}=25$; $dn_{1}=3.6$; ${dn}_{2}=3.6$; $x_{0}$ represents the longitudinal position of the vehicle at the current moment.

**Figure 4:**
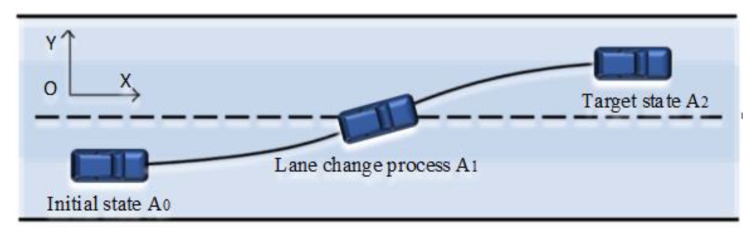
Schematic diagram of obstacle-free lane-changing trajectory.

The lane-change trajectory is shown in Figure 4. Currently, the widely used trajectory is the sinusoidal function-based lane-change trajectory, which is easy to calculate and has excellent smoothness characteristics.

This trajectory planning can simulate the smooth transition of steering and longitudinal control in actual lane-change scenarios. The expressions for the desired lateral position $y_{r}$ and yaw angle $\psi_{r}$ of this lane-change condition are given as follows [32]:

(50) $$y_{r}=\frac{4}{2\pi }\left(\pi +\frac{2\pi }{100}\left(x_{0}-50\right)+\sin\left(\frac{2\pi }{100}\left(x_{0}-50\right)\right)\right)\;$$

(51) $$\psi_{r}=\frac{4}{2\pi }\left(\frac{2\pi }{100}+\frac{2\pi }{100}\cos\left(\frac{2\pi }{100}\left(x_{0}-50\right)\right)\right)$$

### 5.2. Simulation Analysis of Hamilton Dissipative Control

**Figure 5:**
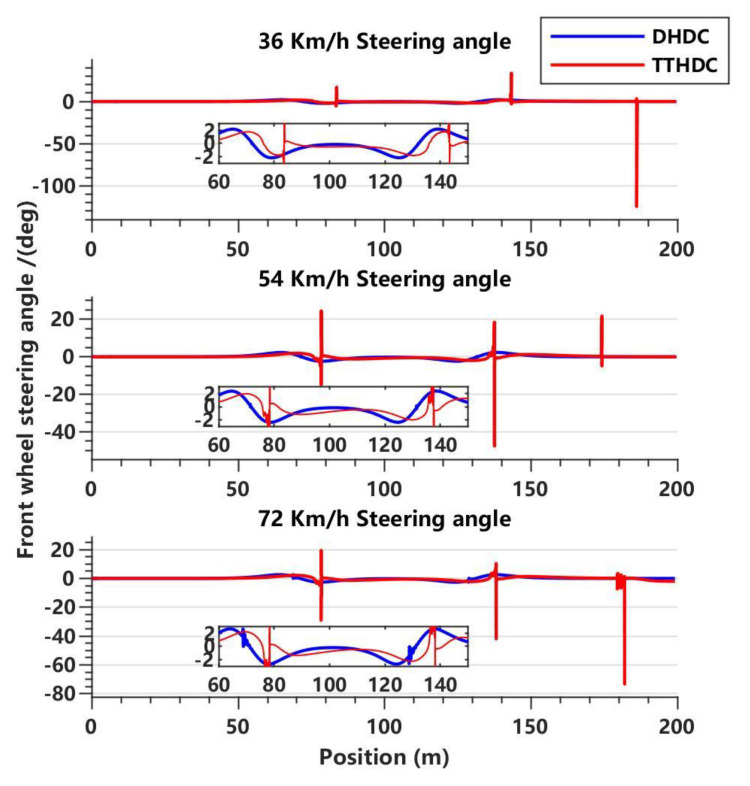
**Trends of** front wheel steering angle (FWSA) for the DHDC and TTHDC at three vehicle speeds.

**Figure 6:**
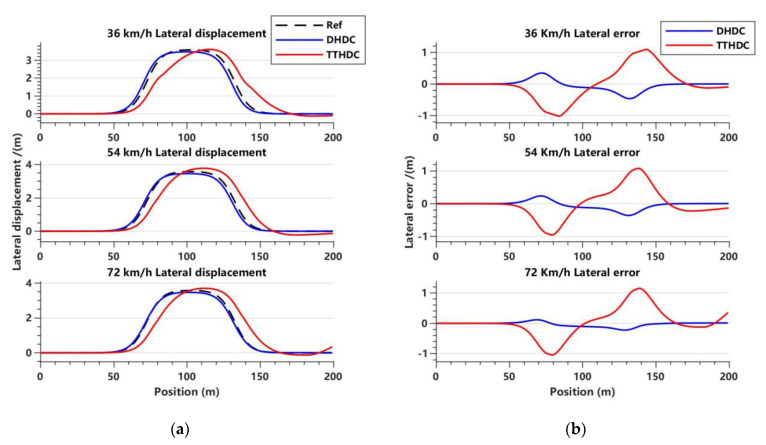
Variations of lateral displacement and lateral error with displacement for the DHDC and TTHDC in the double lane road condition. (**a**) Lateral displacement tracking effect at three vehicle speeds; (**b**) lateral error at three vehicle speeds.

**Figure 7:**
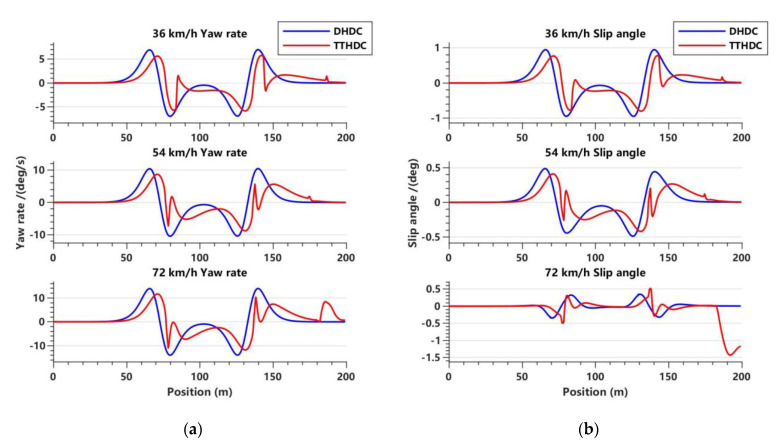
Variations in lateral yaw rate and slip angle with displacement for the DHDC and TTHDC in the double lane road condition. (**a**) Lateral yaw rate variation trend at three vehicle speeds; (**b**) slip angle variation trend at three vehicle speeds.

In this section, the dynamics Hamilton dissipative controller (DHDC) and trajectory-tracking Hamilton dissipative controller (TTHDC), designed as described above, are used to carry out trajectory tracking control of the vehicle. It should be noted that in the vehicle dynamics control system, due to the simplification of the vehicle model, there may be undercompensation in the FWSA. Therefore, based on Equation (35), the steering compensation gain parameter $\epsilon_{1}$ is introduced, such that $\mu_{11}^{\prime }=\mu_{1}^{\prime }/\epsilon_{1}$. In the vehicle trajectory-tracking control system, there may be internal disturbances in the vehicle systems that cause local oscillations during the control processes. Therefore, based on Equation (22), the antidisturbance gain parameter $\epsilon_{2}$ is introduced, such that $\mu_{11}=\mu_{1}/\epsilon_{2}$. Through extensive simulation data, it has been found that regardless of the value of $\epsilon_{1}$, the DHDC cannot achieve satisfactory control performance at three different vehicle speeds. Therefore, we set $\epsilon_{1}$ to be 0.008, 0.04, and 0.068, corresponding to vehicle speeds of 36 km/h, 54 km/h, and 72 km/h, respectively. The vehicle trajectory-tracking control system may better describe the motion state of the vehicles, so the TTHDC solves the problem of the poor adaptability of the DHDC. Therefore, $\epsilon_{2}$ is set to 2.7 for all three different vehicle speeds. Figure 5, Figure 6 and Figure 7 show the simulation results of the DHDC and TTHDC in the double lane-change road condition.

Based on the variation of the FWSA in Figure 5, it can be observed that the DHDC demonstrates a relatively stable control performance. At a higher speed of 72 km/h, slight shaking occurs during the vehicle’s steering processes. On the other hand, the TTHDC occasionally shows noticeable shaking during straight-line driving or steering of the vehicle. This could be due to disturbances present in the internal system of the vehicle-trajectory tracking model. In addition, the higher the speed, the more pronounced the shaking during steering, resulting in poorer control performance.

Based on the lateral displacement tracking performance and the variation of the lateral error in Figure 6a,b, it can be observed that both the DHDC and TTHDC are capable of tracking the reference trajectory. However, the DHDC shows a better tracking performance with higher accuracy. It should be noted that the steering compensation gain parameter chosen by the DHDC varies for different vehicle speeds. Therefore, although the DHDC achieves high tracking accuracy, its adaptability to different conditions is poor, and it may require a combination of other control methods to achieve better control performance. Under the influence of internal disturbances in the system, the trajectory-tracking performance of the TTHDC is not ideal, although the tracking accuracy is higher than the general accuracy requirements. The tracking performance is less stable at 36 km/h and 72 km/h than at 54 km/h, suggesting that the vehicle speeds may have some influence on its trajectory-tracking control.

Based on the variations of yaw rate and slip angle shown in Figure 7a,b, their trends are similar. Under different gain parameters, the DHDC shows good stability. In other words, as long as the parameters are properly chosen, the DHDC can stably track the vehicle’s trajectory. As mentioned earlier, the TTHDC has poor stability, and both the yaw rate and slip angle show varying degrees of shaking. At 72 km/h, it may even affect the vehicle’s normal straight-line driving and pose unnecessary safety risks.

Overall, the DHDC with adjusted steering compensation gain can effectively control the vehicle’s trajectory tracking. With an appropriate choice of gain parameter, it can ensure better trajectory tracking accuracy and stability. On the other hand, the TTHDC with increased antidisturbance gain provides some suppression of internal disturbances in the system, but it still exhibits unstable control. Both the tracking accuracy and stability fall short of general control requirements, making its trajectory-tracking control performance less than ideal.

### 5.3. Comparison and Analysis of Generalized Hamilton Robust Controller (GHRC) Simulation

In this section, a simulation of the intelligent vehicle trajectory-tracking control is carried out based on the GHRC designed in Section 4. To comprehensively test the performance of this controller, simulations are conducted for both the double lane-change trajectory and lane-change trajectory at three different vehicle speeds. In addition, the SMC and LQR controllers are selected for comparison. The GHRC has good robustness, which eliminates the need for additional control parameters. Table 1 shows the GHRC parameter values.

#### 5.3.1. Double Lane-Change Scenarios

**Figure 8:**
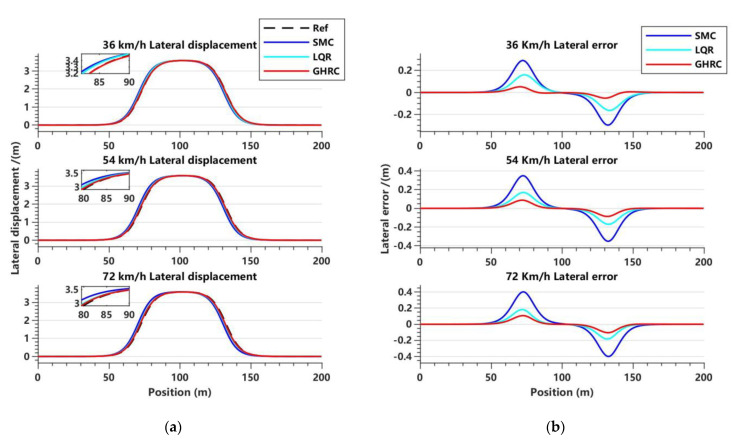
Variations in lateral displacement and lateral error with respect to displacement in the double-lane road condition. (**a**) Lateral displacement tracking effect at three vehicle speeds; (**b**) lateral error at three vehicle speeds.

Figure 8a,b illustrate the variations in lateral displacement and lateral displacement error of each controller at different speeds.

Meanwhile, Table 2 and Table 3 list the peak lateral error and root mean square error (RMSE) of each controller at different speeds. These graphs and tables provide a clear understanding of the performance of different controllers at different speeds, which serves as a reference for further research and optimization.

Based on Figure 8a,b, it is evident that the GHRC performs better when tracking the reference trajectory of the double lane-change maneuver at the three different vehicle speeds. Based on the data analysis results shown in Table 2 and Table 3, it can be inferred that the lateral displacement peak error of the GHRC is 0.0514 m at a vehicle speed of 36 km/h, which is 82.27% and 67.90% lower than that of the SMC and LQR controllers, respectively. This shows that the GHRC has higher tracking accuracy. For vehicle speeds of 54 km/h and 72 km/h, the GHRC reduces the lateral displacement peak error by 41.24% to 75.05% compared to the SMC and LQR controllers, while the error fluctuation optimization effect is from 43.28% to 75.68%. Therefore, it can be concluded that the GHRC has a good optimization performance in terms of trajectory tracking accuracy and trajectory smoothness under a double lane-change maneuver for low, medium, and medium–high speed conditions.

**Figure 9:**
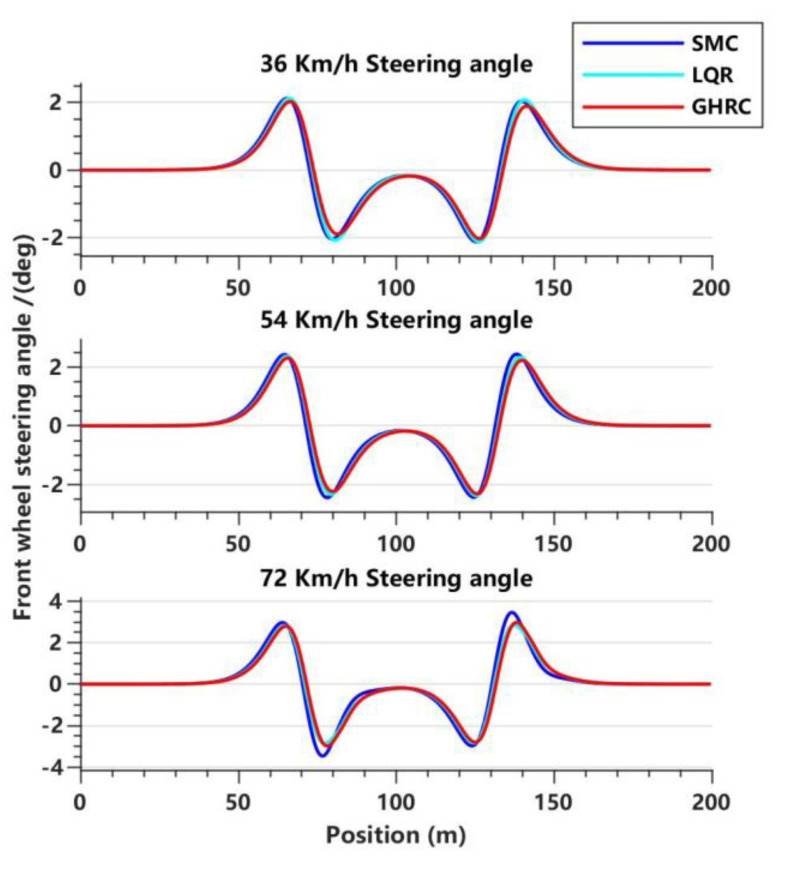
**Comparison of** FWSA for three controllers in the double lane-change maneuver at three vehicle speeds.

Figure 9 illustrates the input of the FWSA by the three controllers at different vehicle speeds. It can be observed that at various vehicle speeds, the GHRC has better control performance on the FWSA than the SMC and LQR control. As the vehicle speed increases, the control effect of the LQR gradually approaches that of the GHRC, and the difference between them is not significant when the speed reaches 72 km/h. Through calculations, it was found that compared with the SMC, the optimization effect of the GHRC gradually increases from 4.53% to 13.87% with the increase in vehicle speed, and the optimization effect of its root mean square (RMS) value is also between 4.20% and 7.67%. This indicates that the GHRC has a better control performance and a smoother FWSA curve.

**Figure 10:**
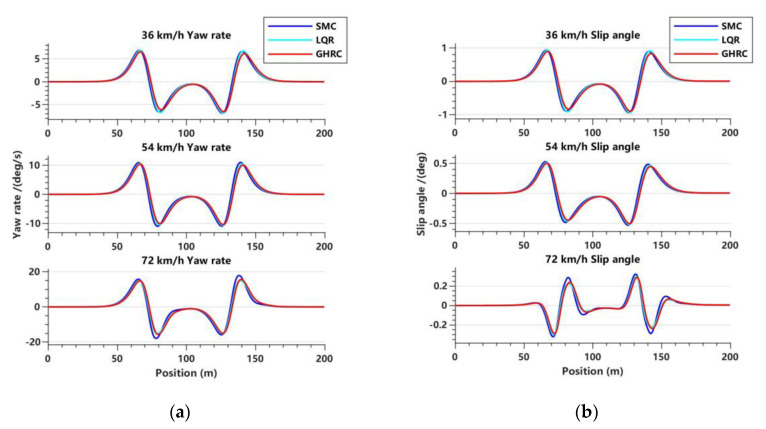
Variations in lateral yaw rate and slip angle with respect to displacement in the double lane road condition. (**a**) Lateral yaw rate variation trend at three vehicle speeds; (**b**) slip angle variation trend at three vehicle speeds.

Figure 10a illustrates a comparison of the lateral yaw rate of intelligent vehicles under different vehicle speeds. The results indicate that all three controllers can maintain the lateral stability of the vehicle at different speeds. It can be observed that the lateral yaw rate amplitudes of the three controllers increase with the increase in vehicle speed. Furthermore, it was found that the lateral yaw rate amplitude of the GHRC was lower than the other two controllers at different speeds, indicating that the Hamilton control method has better stability.

Figure 10b presents the centroid sideslip angles of the three controllers at different vehicle speeds, which indicates that the difference among them is not significant, and their peak values are far below the limit of 8.91°. At the speed of 72 km/h, the centroid sideslip angles of the three controllers show small fluctuations within a reasonable range. Comparatively, the GHRC shows less fluctuation and a better overall performance. Therefore, compared with the other two controllers, the GHRC not only applies to medium and low speeds, but also provides a better tracking control performance for the vehicle at medium–high speeds.

In summary, the results have confirmed the good accuracy and stability of the GHRC in vehicle-trajectory tracking under double lane-change tests, with better overall performance than the SMC and LQR controllers. At a speed of 72 km/h, although the LQR controller’s performance is relatively close to that of the GHRC in some aspects, there is a significant difference in the lateral error, with the GHRC able to achieve an optimization effect of 43.28% over the LQR controller.

#### 5.3.2. Lane-Changing Scenarios

**Figure 11:**
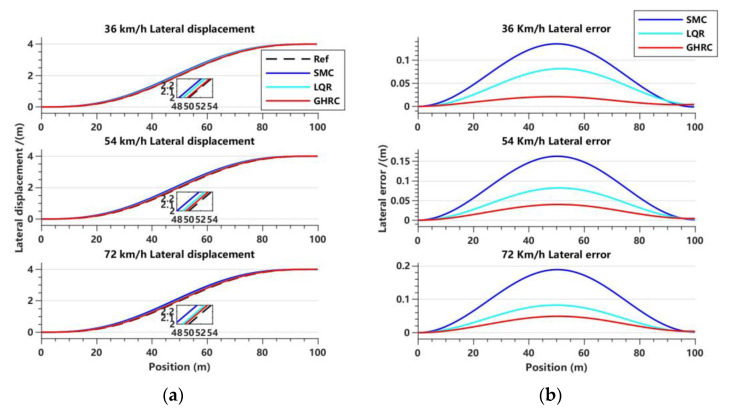
Variations in lateral displacement and lateral error with respect to displacement in the lane-changing condition. (**a**) Lateral displacement tracking effect at three vehicle speeds; (**b**) lateral error at three vehicle speeds.

Figure 11 illustrates the variations of lateral displacement and lateral displacement error of each controller at different speeds.

Meanwhile, Table 4 and Table 5 list the peak lateral error and RMSE of each controller at different speeds.

From the trajectory tracking effect in Figure 11a and the comparison of lateral error in Figure 11b, it can be observed that the GHRC has higher tracking accuracy, a smoother curve trajectory, and better robustness during lane change under three different vehicle speeds. Analysis of the data in Table 4 and Table 5 also shows that the peak lateral displacement error of the GHRC is 0.021 m at a vehicle speed of 36 km/h, which is reduced by 84.44% and 74.23% compared to the SMC and LQR controllers, respectively. The lateral displacement RMSE is 0.0133 m, and the optimization effect is 83.92% and 73.56% for the SMC and LQR controllers, respectively. When the vehicle speed is 54 km/h and 72 km/h, the optimization effect of the peak lateral displacement error of the GHRC is between 40.56% and 75.38% compared to the SMC and LQR controllers, and the optimization effect of the error fluctuation is between 40.16% and 74.95%. This indicates that the GHRC has good robustness while ensuring tracking accuracy, and the effect is more evident at slower speeds.

**Figure 12:**
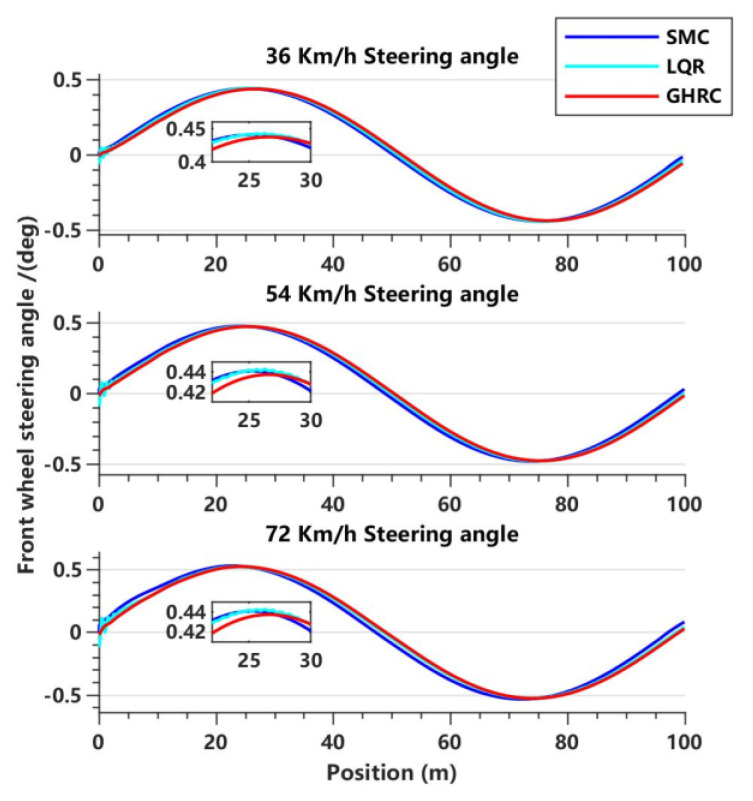
**Comparison of** FWSA for three controllers in the lane-change maneuver at three vehicle speeds.

Figure 12 shows the variation in the FWSA curve for three controllers at different vehicle speeds. It can be observed from the figure that the LQR controller shows a slight oscillation in controlling the FWSA, which may be due to the unsuitability of the LQR parameters for this operating condition. Furthermore, this indicates that the adaptability of the LQR controller to the operating condition is poor under the same parameters, and therefore online parameter tuning based on road condition information may be necessary. The GHRC still shows better performance than the SMC. Although the optimization effect of FWSA-RMS value is only 0.80~1.04%, it can be seen that with the increase in vehicle speed, the control effect becomes better and better, which can better ensure the safety of the vehicle at medium and high-speed driving.

**Figure 13:**
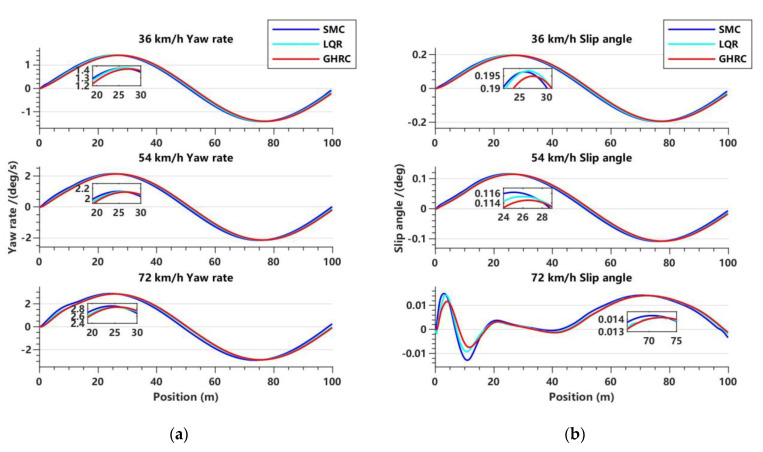
Variations of lateral yaw rate and slip angle with respect to displacement in the lane-changing condition. (**a**) Lateral yaw rate variation trend at three vehicle speeds; (**b**) slip angle variation trend at three vehicle speeds.

Figure 13a compares the performance of the three controllers in terms of the lateral yaw rate of intelligent vehicles at different speeds. The results indicate that the lateral yaw rate of the three controllers shows slight fluctuations with increasing speed. However, the GHRC has smaller fluctuations compared to the other two controllers. Overall, the lateral yaw rate curves of the three controllers show little difference in trend. But, as shown in the local magnified image, the lateral yaw rate amplitude of the GHRC method is smaller, indicating that it is more stable.

Figure 13b compares the centroid sideslip angle curves of the three controllers at different vehicle speeds. It can be observed that the trends of the centroid sideslip angles for all three controllers are similar, with the GHRC showing a slightly better performance compared to the other two controllers. At a speed of 72 km/h, all three controllers show reasonable fluctuations in the centroid sideslip angle. Compared to the SMC and LQR controller, the GHRC shows relatively smaller fluctuations, with optimization effects on the centroid sideslip angle RMS of 5.95% and 2.47%, respectively.

Using the real-time synchronization function in MATLAB, we obtained the average computation time for each controller in a double-shifted trajectory at different vehicle speeds, as shown in Table 6.

In the literature [11], the average computation time of the controllers designed using the forward Eulerian and backward Eulerian methods for a double lane-change trajectory is mentioned. Specifically, the average computation times of the controllers designed using the forward Eulerian and backward Eulerian methods are 0.0084 s and 0.0178 s, respectively, when the speed is 40 km/h; and 0.0084 s and 0.008 s, respectively, when the speed is 60 km/h. It can be seen from Table 6 that there is not much difference in the average computation times of the controllers at the same speed. As the vehicle speed increases, the computation time gradually decreases, which is consistent with the results in the literature [11]. It is worth noting that the computation time of the controllers designed in this paper does not differ much from the data in the literature [11], and even improves slightly. Therefore, from these data of average computation time, it can be seen that each of the controllers designed in this paper has good real-time performance.

In summary, the comparative analysis of simulation results between the GHRC, SMC, and LQR controller verifies that the GHRC algorithm has good adaptability to simulated driving conditions, superior overall performance, strong robustness, and better tracking control accuracy. Although the LQR controller shows only minor differences in terms of the FWSA, yaw rate, and center of mass sideslip angle compared to the GHRC in the lane change and double lane change scenarios at 72 km/h, the GHRC has better adaptability to the scenarios and significantly lower lateral error, resulting in an optimization effect ranging from 40.56% to 84.44%.

## 6. Conclusions

1. This paper first proposes an intelligent vehicle trajectory-tracking control strategy based on the generalized Hamilton theory. This strategy combines the vehicle-dynamic model with the trajectory tracking model to establish a trajectory-tracking control system. By employing orthogonal decomposition and control switching methods for the first time, two Hamilton dissipative controllers are designed. As a result, feedback-dissipative Hamilton realizations are obtained for both the dynamic control system of intelligent vehicles and the trajectory-tracking control system, enabling the self-stabilizing control of intelligent vehicles.
2. By utilizing the derived dissipative Hamilton realization of the intelligent vehicle’s trajectory-tracking control system, further combined with the Hamilton robust control approach, the FWSA is employed as the control output, while the lateral velocity error and yaw angular velocity error serve as the state inputs. A robust controller based on the generalized Hamilton principle is designed. Furthermore, stability analysis is performed using the Lyapunov function and LaSalle invariance principle, which verifies the stability of the GHRC.
3. In the co-simulation model developed in this paper, the three controllers are used separately to verify the control effects of the vehicle-trajectory tracking. The comparative analysis showed that both the DHDC and TTHDC effectively control the trajectory tracking of intelligent vehicles. However, due to model inaccuracy or the presence of internal system disturbances, problems such as low tracking accuracy and poor stability arise. On the other hand, the GHRC not only addresses the problems of DHDC and TTHDC, but also demonstrates improved trajectory tracking precision and robustness compared to the SMC and LQR control methods in double lane-change and lane-change scenarios. The peak lateral position error can be reduced by up to 84.44% and the RMS values of various state curves have also been reduced with a maximum reduction in fluctuation of 11.99%.
4. In future research, the scope of this paper will be further extended to cover more practical issues. These issues include a more detailed consideration of the uncertainty in vehicle modeling and the effects of various disturbances in the real environment on vehicle motion. We will also investigate the effects of factors such as vehicle time delay and actuator saturation on the system. In addition, we plan to further improve and optimize the control algorithm proposed in this paper and consider conducting real vehicle experiments to provide some new ideas and methods for vehicle-trajectory tracking control in practice.

## Figures and Tables

**Table 1:** Controlled vehicle parameters.

Name/Unit	Symbolic Representation	Numerical Value
Vehicle mass/kg	m	1412
Front wheels side deflection stiffness/(N/rad)	$K_{f}$	−57,500
Rear wheels side deflection stiffness/(N/rad)	$K_{r}$	−57,500
Moment of inertia/(kg·m^2^)	$I_{z}$	1536
Distance from front axle to center of mass/m	a	1.015
Distance from rear axle to center of mass/m	b	1.895
GHRC weighting coefficients	r	0.05
GHRC suppression level	λ	8

**Table 2:** Peak lateral displacement error in the double shift line condition.

Vehicle Speed/(km/h)	SMC/m	LQR/m	GHRC/m	Optimization Effect 1	Optimization Effect 2
36	0.2899	0.1601	0.0514	82.27%	67.90%
54	0.3479	0.1686	0.0868	75.05%	48.52%
72	0.4027	0.1821	0.107	73.43%	41.24%

**Table 3:** Root mean square error (RMSE) of lateral displacement in the double shift line condition.

Vehicle Speed/(km/h)	SMC/m	LQR/m	GHRC/m	Optimization Effect 1	Optimization Effect 2
36	0.1078	0.0622	0.0177	83.58%	71.54%
54	0.1213	0.0607	0.0295	75.68%	51.40%
72	0.1476	0.0670	0.038	74.25%	43.28%

**Table 4:** Peak lateral displacement error in the lane-changing condition.

Vehicle Speed /(km/h)	SMC/m	LQR/m	GHRC/m	Optimization Effect 1	Optimization Effect 2
36	0.135	0.0815	0.021	84.44%	74.23%
54	0.1629	0.0821	0.0401	75.38%	51.16%
72	0.1891	0.0826	0.0491	74.03%	40.56%

**Table 5:** RMSE of lateral displacement in the lane-changing condition.

Vehicle Speed/(km/h)	SMC/m	LQR/m	GHRC/m	Optimization Effect 1	Optimization Effect 2
36	0.0827	0.0503	0.0133	83.92%	73.56%
54	0.0998	0.0506	0.025	74.95%	50.59%
72	0.1159	0.0508	0.0304	73.77%	40.16%

**Table 6:** Controller computation time.

Vehicle Speed/(km/h)	DHDC/s	TTHDC/s	GHRC/s
36	0.014	0.012	0.011
54	0.007	0.007	0.008
72	0.005	0.005	0.005

## Data Availability

Not applicable.
